# Novel Modification of Integrated Optimization Method for Sensor’s Communication in Wi-Fi Public Networks

**DOI:** 10.3390/s24051395

**Published:** 2024-02-21

**Authors:** Sergey Kozlov, Elena Spirina

**Affiliations:** Radioelectronic and Telecommunication Systems Department, Kazan National Research Technical University Named after A. N. Tupolev-KAI, Kazan 420111, Russia; easpirina@kai.ru

**Keywords:** intra-system interference, sensor lifetime, heterogeneous networks, IP networks integrated optimization method, novel modification of the collective dynamic routing method

## Abstract

A novel modification of IP networks integrated optimization method for heterogeneous networks, for example, the seamless Wi-Fi network serving simultaneously mobile users and wireless sensors, has been developed in this article. The mutual influence of signal reception, frequency-territorial planning, and routing procedures in heterogeneous networks have been analyzed in the case of simultaneous data transmission by both mobile users and wireless sensors. New principles for the listed procedures interaction and the basic functions for their describing are formulated. A novel modification of the integrated optimization method and its algorithm have been developed. The developed method's effectiveness has been analyzed for the IEEE 802.11ax network segment. Its result showed that the network load was decreased by an average of 20%, the data rate over the network as a whole increased for users and sensors by an average of 25% and 40%, respectively, and the sensors’ lifetime increased by an average of 20% compared to the novel modification of the Collective Dynamic Routing method.

## 1. Introduction

With the development of the IoT, the requirements for networks that provide data transmission from wireless sensors that have strict restrictions on energy consumption have sharply increased.

Today, dedicated networks based on special technologies LoRaWAN [1], SigFox [2], Bluetooth [3], etc., are used to transmit data by wireless sensors, as well as heterogeneous networks using Wi-Fi [4], LTE [5], 5G NR, and their combinations that simultaneously serve mobile users and wireless sensors.

In [6], for a seamless IEEE 802.11ax network simultaneously serving mobile users and sensors, a novel modification of the collective dynamic routing (CDR) method was developed, which allows for reducing the sensors' energy consumption while maintaining high data rates and short data delivery times for mobile users.

However, the CDR method was developed within the framework of the integrated optimization method (IOM) of IP networks [7], which makes it possible to achieve additional improvement in these networks' characteristics through joint procedures’ optimization of signal reception [8], frequency-territorial planning (FTP) [9] and routing [10] due to the comprehensive reduction of the intra-system interference influence.

Currently, improving the network characteristics by reducing the intra-system interference influence is carried out in three main areas separately. To combat intra-system interference, optimal and quasi-optimal signal reception procedures are widely used [11,12,13,14,15]. To reduce the intra-system interference level, FTP procedures are used [16,17,18]. To reduce the intra-system interference flow, routing procedures are used [6,19,20].

However, the procedures for signal reception, FTP, and routing are interconnected, so their separate optimization does not guarantee the potential characteristics achievement of the network as a whole.

The IOM of IP networks proposed in [7] and described in [8,9,10,21] involves optimizing parameters for each route from a multitude of valid routs set $\left\{w\right\}$, including both one-dimensional and multidimensional routes, and informing network nodes about the optimal routes set according to which the data will be transmitted. However, valid route sets $\left\{w\right\}$ and optimal route sets are formed only in the classical CDR method, which, as shown in [6], is not applicable in heterogeneous Wi-Fi networks simultaneously serving mobile users and sensors. To apply the IOM in such networks, it is necessary to use a novel modification of the CDR method presented in [6]. For this modification, the use of the procedures interaction set principles out in [7,8,9,10,21] is not possible.

Therefore, an urgent task is to develop a novel modification of IOM for these networks. The article presents a novel modification of procedures and principles of their interaction for the IOM, taking into account the specifics of the novel modification of the CDR method and heterogeneous Wi-Fi networks simultaneously serving mobile users and sensors. For novel modification of IOM, a new algorithm has been developed, and changes have been made to the optimization criteria and restrictions. Also, the accounting of sensor battery charge both when optimizing network parameters and when generating optimal routes is added.

## 2. Existing Signal Reception, FTP, and Routing Methods Review

The signals reception procedure task is to ensure the data transmission through communication channels (CCs) at the highest data rate, provided that the error probability is not greater than a specified one. To do this, the signals reception procedure uses optimal and quasi-optimal reception algorithms.

Thus, the works [12,13] describe optimal algorithms that provide an increase in data rate by eliminating the transmission of a cyclic prefix in a channel with memory when using reception as a whole. One example of similar quasi-optimal algorithms is the Zero Forcing algorithm [14].

In [15], an analysis of the noise immunity of reception algorithms against a background of non-Gaussian noise is carried out and it is proposed to use nonlinearities to correct the noise distribution.

However, the algorithms presented in the reviewed works do not take into account information about the number of currently transmitted signals and their parameters, available in FTP and routing procedures.

To ensure sufficient signal strength for data transmission and to minimize the levels of intra-system interference created by signals from other network devices, the FTP procedure is used.

In [17], it is proposed to jointly optimize the coordinates of access points (APs), their parameters (transmission power, azimuth, radiation patterns, etc.), and channel distribution to maximize the coverage area and minimize intra-system interference level.

In [18], it is proposed to use a heterogeneous network (HetNet) with small cells of LTE and Wi-Fi standards to improve the efficiency of using a limited frequency resource.

However, when minimizing the intra-system interference levels, the considered works do not take into account information about the number of simultaneously transmitted signals and the degree of signal influence from other devices on the data rate available in the procedures for routing and signal reception.

A detailed review of routing methods is provided in [6]. In it, a novel modification of the CDR method has been developed for simultaneous servicing of mobile users and wireless sensors. However, for its effective use, it is necessary to know the list of accessible CCs and the data rates through them, available in FTP and signals reception procedures.

## 3. Relationship Analysis between Procedures of the Signals Reception, FTP, and Routing

**Figure 1:**
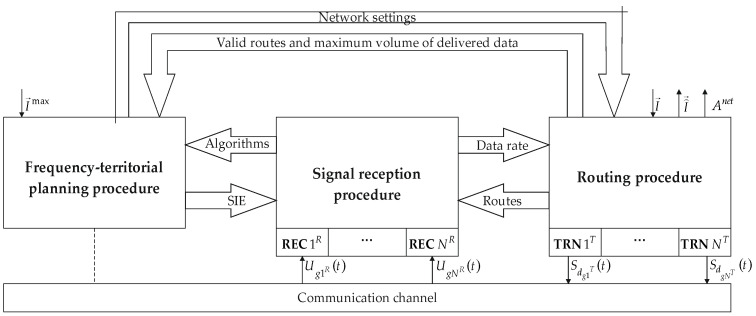
Signals reception, FTP, and routing procedures interconnections diagram.

The relationship between the signals reception, FTP, and routing procedures, schematically presented in Figure 1, was considered for a network comprising $N^{T}$ transmitting and $N^{R}$ receiving nodes.

This network’s task is to deliver a current volume $\vec{I}$ accumulated at a certain time interval $T^{I}$ with an error probability no more than $P_{\max}^{Er}$ using a finite valid routes set $\left\{w\right\}$.

Data is transmitted over the network using a valid routes set $\left\{w\right\}$. It is formed in the routing procedure based on the parameters specified in the network frequency–territorial plan, which is determined at the FTP stage.

The optimal routes set for data transmission is selected at the OSI model network level based on some optimization criteria. Currently, minimizing the data delivery time criterion is widely used. However, in existing routing methods, the data (packets) delivery time along a route $w_{g}$, $g=\bar{1,G}$ is determined without taking into account the mutual influence of the CCs included in it.

The choice of one or another route leads to the simultaneous use of certain transmitting nodes set for data transmission. The signals $S_{d_{gn^{T}}}\left(t\right)$ of these transmitting nodes under conditions of CCs' mutual influence create an intra-system interference flow. Its flow forms a certain signal-interference environment (SIE), depending on the chosen route.

Based on the SIE, the modulation type at the OSI model's physical level and the data encoding scheme at the OSI model channel level are selected. These modulation types and coding schemes determine the channel data rate. This rate and the media access method latency determine the data delivery time.

This means that the route choice influences the CIE in the CC, the channel data rate, and the data delivery time.

Thus, the data flow distribution optimization at the network level should be carried out, taking into account the intra-system interference flow influence that occurs when transmitting data along a particular selected route. Therefore, to effectively reduce the impact of the intra-system interference flow, the routing method must ensure the three OSI model layers interaction: network, channel, and physical.

Allowable data rates are determined in the signals reception procedure depending on the SIE in the CC, that characterized by the oscillation $U_{gn^{R}}\left(t\right)$ parameters at the receiving nodes input and the used reception algorithms.

The maximum efficiency is achieved when optimal reception algorithms and adequate models for describing real SIE are used. These algorithms ensure, for example, minimizing the error probability when receiving transmitted data. However, in the case under consideration, the optimal reception algorithm task can be formulated as maximizing the data rate when an error probability is no more than $P_{\max}^{Er}$.

In real conditions, SIE has high complexity and dynamism, especially mobile networks, which leads to the objective complexity of the models describing it. This circumstance, in turn, coupled with the unknown number of signals present at the input of the receiver, leads to high complexity in the algorithms implementation and devices synthesized on the basis of these models.

In addition, using complex models and algorithms generally requires a fairly accurate determination of a large number of their parameters. In real networks, estimating these parameters with the required reliability is quite complex and often impossible.

However, the use of information available in FTP and routing procedures when receiving signals makes it possible to change the existing methodology for synthesizing optimal reception algorithms by implementing “control” of SIE in order to reduce the uncertainty existing in the CC.

Thus, taking into account the route information from the routing procedure in the signal reception procedure allows for reducing the SIE uncertainty by determining the number of signals used for data transmission. Taking this same information into account in the FTP procedure allows us to evaluate and optimize the SIE for each of the valid routes to reduce the intra-system interference impact. In this case, each route is characterized by its own best SIE, for which the achievement of the highest data rates will be ensured by its own reception algorithm.

Taking into account in the routing procedure information about data rates available in the signals reception procedure will allow the formation of optimal routes set according to the criterion of minimizing data delivery time. Information about the optimal routes set will provide an additional reduction in SIE uncertainty for the signals reception procedure.

In addition, taking into account information about data rates will allow the routing procedure to estimate the real volumes of data being delivered $\vec{\overset{⌢}{I}}$ and the network load $A^{net}$. Their use in the FTP procedure allows you to check whether the network parameters meet the requirements: the ability to deliver the required volumes of data being delivered ${\vec{I}}^{\max}$.

Taking into account information about the selected reception algorithm from the signals reception procedure and information about the valid routes set $\left\{w\right\}$, the real volumes of data being delivered $\vec{\overset{⌢}{I}}$ and network load $A^{net}$ from the routing procedure, as well as the required volumes of data being delivered ${\vec{I}}^{\max}$ in the FTP procedure will allow for optimizing SIE parameters for each of the routes taking into account the selected reception algorithm.

Thus, the procedures for signal reception, FTP, and routing mutually influence each other. Mutual accounting of information generated in each of them will make it possible to comprehensively reduce the intra-system interference impact, and therefore improve the network characteristics as a whole. For this purpose, the IOM of IP networks was developed. This method makes it possible to implement the listed procedures interaction by jointly considering their parameters, forming a unified network parameters set $V^{u}$.

However, since the existing IOM of IP networks does not take into account the networks with sensor features, the urgent task is to develop a novel modification of IOM for a seamless Wi-Fi network that simultaneously serves mobile users and sensors.

## 4. Principle of Interaction between Signals Reception, FTP, and Routing Procedures in a Seamless Wi-Fi Network with Sensors

An arbitrary seamless Wi-Fi network segment with sensors consisting of a backbone router (BR), $N^{I}$ APs, $N^{U}$ users, and $N^{S}$ sensors is presented in [6]. The task of an arbitrary seamless Wi-Fi network segment is to transmit data from the BR to all users and sensors and from all users and sensors to the BR accumulated over the data accumulation interval $T^{I}$ with an error probability of no more than the acceptable value $P_{\max}^{Er}$. The optimization criteria are minimizing data delivery time and maximizing sensor lifetime.

As in [6], the option when data transmission between BR and users, as well as between BR and sensors, is carried out at different times is considered. Because the current volumes of data delivered are divided into four vectors $\vec{I}=\left({\vec{I}}^{DU},{\vec{I}}^{UU},{\vec{I}}^{DS},{\vec{I}}^{US}\right)$, respectively, and the network load is the sum of the corresponding loads $A^{net}=A^{netDU}+A^{netUU}+A^{netDS}+A^{netUS}$, where DU and DS indicate data transfer in the downlink from BR to users and sensors and UU and US indicate data transfer in the uplink from users and sensors to BR, respectively.

To reduce the sensors’ energy consumption, the period of their polling was increased to a value $T^{S}$ equal to the integer number of intervals $T^{I}$ by dividing the sensors into groups, each of which is serviced in its own $T^{I}$ interval.

**Figure 2:**
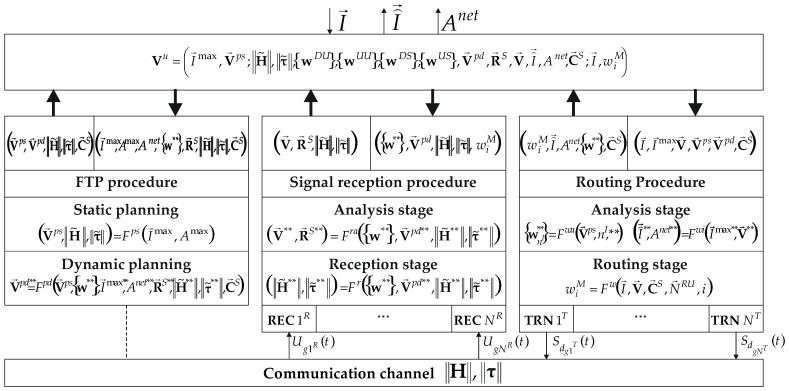
Interaction principle between procedures of signals reception, FTP and routing, where** are DU, UU, DS, US respectively.

The principle of interaction between signal reception, FTP, and routing procedures using unified parameters set $V^{u}$ for a seamless Wi-Fi network with sensors is illustrated in Figure 2.

The first stage of designing a seamless Wi-Fi network with sensors is the FTP procedure. In it, network parameters are determined that ensure delivery of the required volumes of data being delivered to ${\vec{I}}^{\max DU}=\left(I_{1}^{\max DU},\ldots ,I_{N^{U}}^{\max DU}\right)$, from ${\vec{I}}^{\max UU}=\left(I_{1}^{\max UU},\ldots ,I_{N^{U}}^{\max UU}\right)$ of all users and to ${\vec{I}}^{\max DS}=\left(I_{1}^{\max DS},\ldots ,I_{N^{S}}^{\max DS}\right)$, from ${\vec{I}}^{\max US}=\left(I_{1}^{\max US},\ldots ,I_{N^{S}}^{\max US}\right)$ of all sensors located in the $S^{net}$ service area. The controlled variables vector for FTP includes a standard parameter set: number of users $N^{U}$, sensors $N^{S}$ and APs $N^{I}$, their location coordinates, frequencies, maximum resource units number of each AP in a given frequency band ${\vec{N}}^{RU}=\left(N_{1^{I}}^{RU},N_{2^{I}}^{RU},\ldots ,N_{N^{I}}^{RU}\right)$, power, suspension heights and azimuths of aerials, their radiation patterns, etc. These parameters determine SIE in network CCs. The parameters included in the vector of controlled variables can be divided into two groups: static ${\vec{V}}^{ps}$, which cannot automatically change during the operation of a seamless Wi-Fi network with sensors, and dynamic ${\vec{V}}^{pd}$, which can change during the operation of this network.

Since SIE in CCs is currently considered an objective reality that does not depend on the functioning of the network, SIE is determined only by the static network parameters vector ${\vec{V}}^{ps}$.

At the same time, the static parameters of a seamless Wi-Fi network with sensors include the number of users $N^{U}$, sensors $N^{S}$ , and APs $N^{I}$, their installation places, frequencies, maximum number of resource units of each AP in a given frequency band ${\vec{N}}^{RU}$, maximum powers, suspension heights, azimuths and radiation patterns of AP antennas. These parameters can only be changed during the network's initial planning or when changing its configuration. For this purpose, the FTP procedure provides a static planning stage. This stage task is to determine the static network parameters vector, providing conditions for the delivery of the required volumes of data being delivered ${\vec{I}}^{\max}=\left({\vec{I}}^{\max DU},{\vec{I}}^{\max UU},{\vec{I}}^{\max DS},{\vec{I}}^{\max US}\right)$. The network load $A^{net}<A^{\max}$ [7] was considered as a criterion for fulfilling this condition, where $A^{\max}$ is the maximum permissible network load value. In real seamless Wi-Fi networks $A^{\max}<1$ due to the need to ensure the service information exchange. The real value $A^{\max}$ is selected for each network depending on the network parameters, its construction methods, etc.

In this case, the static network planning parameters are determined based on the required volumes of data being delivered ${\vec{I}}^{\max}$ and the maximum network load $A^{\max}$:

(1) $$\left({\vec{V}}^{ps},\left\|\tilde{H}\right\|,\left\|\tilde{\tau }\right\|\right)=F^{ps}\left(\;{\vec{I}}^{\max},A^{\max}\right),$$

where $F^{ps}$ is the FTP procedure static network planning function and $\left\|\tilde{H}\right\|$ and $\left\|\tilde{\tau }\right\|$ are the block matrices estimates of the transfer and delay.

An example of implementing the static planning function for the company ZAO Torus-Volga Wi-Fi network segment at a stadium in Kaliningrad city is given in [9].

They are characterized by the CCs parameters and consist of attenuation coefficients ${\tilde{h}}_{n^{R}n^{T}}$ and delay ${\tilde{\tau }}_{n^{R}n^{T}}$ estimates when propagating a signal between transmitting and receiving nodes, respectively:

(2) $$\left\|\tilde{H}\right\|=\left(\begin{array}{cccc} \left\|{\tilde{H}}^{DU}\right\| & 0 & 0 & 0\\ 0 & \left\|{\tilde{H}}^{UU}\right\| & 0 & 0\\ 0 & 0 & \left\|{\tilde{H}}^{DS}\right\| & 0\\ 0 & 0 & 0 & \left\|{\tilde{H}}^{US}\right\| \end{array}\right),$$

(3) $$\left\|\tilde{\tau }\right\|=\left(\begin{array}{cccc} \left\|{\tilde{\tau }}^{DU}\right\| & 0 & 0 & 0\\ 0 & \left\|{\tilde{\tau }}^{UU}\right\| & 0 & 0\\ 0 & 0 & \left\|{\tilde{\tau }}^{DS}\right\| & 0\\ 0 & 0 & 0 & \left\|{\tilde{\tau }}^{US}\right\| \end{array}\right),$$

where $\left\|{\tilde{H}}^{DU}\right\|$, $\left\|{\tilde{H}}^{UU}\right\|$, $\left\|{\tilde{H}}^{DS}\right\|$, $\left\|{\tilde{H}}^{US}\right\|$, $\left\|{\tilde{\tau }}^{DU}\right\|$, $\left\|{\tilde{\tau }}^{UU}\right\|$, $\left\|{\tilde{\tau }}^{DS}\right\|$, $\left\|{\tilde{\tau }}^{US}\right\|$ are estimates of the corresponding transfer and delays matrices.

In a seamless Wi-Fi network with sensors, data to users in the current volume ${\vec{I}}^{DU}=\left(I_{1}^{DU},\ldots ,I_{N^{U}}^{DU}\right)$ and from users in the current volume ${\vec{I}}^{UU}=\left(I_{1}^{UU},\ldots ,I_{N^{U}}^{UU}\right)$, as well as data to sensors in the current volume ${\vec{I}}^{DS}=\left(I_{1}^{DS},\ldots ,I_{N^{S}}^{DS}\right)$ and from sensors with the current volume ${\vec{I}}^{US}=\left(I_{1}^{US},\ldots ,I_{N^{S}}^{US}\right)$ can be delivered using subsets of one-dimensional routes $\left\{w_{n^{I}}^{DU}\right\}$, $\left\{w_{n^{I}}^{UU}\right\}$ for users and subsets of one-dimensional routes $\left\{w_{n^{I}}^{DS}\right\}$, $\left\{w_{n^{I}}^{US}\right\}$ for sensors. These subsets are determined at the analysis stage in the routing procedure based on a vector of static planning parameters ${\vec{V}}^{ps}$:

(4) $$\left\{w_{n^{I}}^{**}\right\}=F^{wa}\left({\vec{V}}^{ps},n^{I},**\right),\;n^{I}=\bar{1,N^{I}},$$

where $F^{wa}$ is the routing procedure function of forming subsets of one-dimensional routes.

The one-dimensional route construction using the graph theory DFS method is considered in [6].

To clarify SIE, taking into account the data transmission specifics in the network, a dynamic planning stage is carried out based on the static planning results. The controlled variables vector for dynamic planning includes only those parameters that can change during the network operation. For seamless Wi-Fi networks with sensors, these parameters include only the transmitting node's power.

According to the robust method given in [6], in the downlink, when data transmission is along a one-dimensional route $w_{gn^{I}}^{D*}$ , all other APs ${nI}^{\prime }\neq n^{I}$ create maximum interference to the route $w_{gn^{I}}^{D*}$, using all resource units to transfer data. In the uplink, when data transmission is along a one-dimensional route $w_{gn^{I}}^{U*}$, the interference is caused only by users (sensors) that create the maximum interference level for the analyzed AP. In the uplink, users (sensors) connected to this AP will use all resource units except for the one used for their connection, and all users (sensors) connected to other APs will use all resource units.

The data rates over CCs in a seamless Wi-Fi network with sensors are determined by SIE, which in turn depends on $\left\|\tilde{H}\right\|$, $\left\|\tilde{\tau }\right\|$, and AP, sensors, and user devices radiated powers along all one-dimensional routes. Since the network’s task is to deliver the vector ${\vec{I}}^{\max}$, then to reduce data delivery time it is necessary that data rates along one-dimensional routes be proportional to ${\vec{I}}^{\max}$.

Therefore, to maintain the CC data rates ratio when changing $\left\|\tilde{H}\right\|$ and $\left\|\tilde{\tau }\right\|$, dynamic planning is used. It consists of adjusting the AP, sensors, and user devices radiated powers along one-dimensional routes.

The main task of adjusting power for sensors is to increase their lifetime. This value depends on the sensor energy consumption and the battery charge. Therefore, when adjusting the sensor's power, the battery charge ${\vec{C}}^{S}=\left(C_{1^{S}}^{S},\ldots C_{N^{S}}^{S}\right)$ must be taken into account. Battery charge $C_{n^{S}}^{S}$ can vary from 1, when the sensor’s battery is fully charged, to 0, when the sensor’s battery is completely discharged. The more the sensor battery drains, the less energy it should consume. The energy consumed by the sensor depends on the consumption of its microprocessor, the receiver, and the transmitter, as well as on its active operation time. If the sensor’s microprocessor consumption is practically independent of the network operation, then the consumption of the sensor receiver and transmitter is directly proportional to the number of frames required to deliver data from (to) the sensor. This number is reduced by minimizing the data delivery time. In addition, the transmitter consumption also depends on its radiated power, which must also be taken into account in the dynamic planning process.

The main task of power regulation for users is to increase the network throughput, which, subject to the given current volumes of data being delivered ${\vec{I}}^{DU}$ and ${\vec{I}}^{UU}$ , is achieved precisely by minimizing data delivery time.

In this case, the result of dynamic planning is a transmitting node powers set for each one-dimensional route included in the dynamic planning parameters vector ${\vec{V}}^{pd**}$:

(5) $${\vec{V}}^{pd**}=\left(P_{1}^{**},\ldots ,P_{G^{**}}^{**}\right)=F^{pd}\left(\;{\vec{V}}^{ps},\left\{w^{**}\right\},{\vec{I}}^{\max**},A^{net**},{\vec{R}}^{S**},\left\|{\tilde{H}}^{**}\right\|,\left\|{\tilde{\tau }}^{**}\right\|,{\vec{C}}^{S}\right),$$

where $\left\{w^{**}\right\}=\overset{N^{I}}{\underset{n^{I}=1}{\cup }}\left\{w_{n^{I}}^{**}\right\}$ is the one-dimensional routes subset for all APs in the uplink and downlink for users and sensors, respectively, $G^{**}=\sum_{n^{I}=1}^{N^{I}}G_{n^{I}}^{**}$ is the number of one-dimensional routes in the subset $\left\{w^{**}\right\}$, $F^{pd}$ is the FTP procedure dynamic planning function, and ${\vec{R}}^{S**}$ is the reception algorithms vectors.

Finding the optimal vector ${\vec{V}}^{pd**}$ value is carried out based on the cyclic coordinate descent method using the following criterion:

(6) $${\vec{V}}^{pd**}=\arg\underset{{{\vec{V}}^{\prime }}^{pd**}}{\max}\left({\vec{I}}^{\max**}\cdot {\vec{V}}^{**}\left({{\vec{V}}^{\prime }}^{pd**}\right)\right).$$

Thus, the output FTP procedure parameters are the static ${\vec{V}}^{ps}$ and dynamic ${\vec{V}}^{pd}=\left({\vec{V}}^{pdDU},{\vec{V}}^{pdUU},{\vec{V}}^{pdDS},{\vec{V}}^{pdUS}\right)$ planning parameters vectors and the transfer $\left\|\tilde{H}\right\|$ and delays $\left\|\tilde{\tau }\right\|$ matrices estimates, together defining SIE of a seamless Wi-Fi network with sensors.

The signals reception procedure should ensure data reception with an error probability of no more than $P_{\max}^{Er}$. This procedure's effectiveness depends on the used reception algorithm $R^{s}$ and SIE, characterized by oscillation parameters at the receiving node's input $U_{gn^{R}}\left(t\right)$.

Oscillation parameters $U_{gn^{R}}\left(t\right)$ determined by the signal parameters of the transmitting nodes $S_{d_{gn^{T}}}\left(t\right)$ and transfer $\left\|H\right\|$ and delay $\left\|\tau \right\|$ matrices, whose assessments $\left\|\tilde{H}\right\|$ and $\left\|\tilde{\tau }\right\|$ were received in the FTP procedure.

To increase the network throughput, the signal reception procedure's main task is to ensure a maximum data rate with the error probability is a more acceptable one.

At the receiving node's inputs for each route, its own SIE is formed. This SIE can vary greatly for different routes. Therefore, to receive data, each route can use its own reception algorithm $R^{S}$. Due to the finiteness of one-dimensional route subsets, the SIEs and the reception algorithms $\left\{R^{S}\right\}$ subsets are finite.

Another signal reception procedure task is to select a reception algorithm that provides the maximum data rate to each of the receiving nodes along each one-dimensional route based on dynamic planning parameters vectors ${\vec{V}}^{pd**}$:

(7) $$V_{g}^{opt**}=\underset{\left\{R^{S}\right\}}{\max}V_{g}^{**}\left(R^{s}\right),$$

(8) $$R_{g}^{S\_opt**}=\arg\underset{\left\{R^{S}\right\}}{\max}V_{g}^{**}\left(R^{s}\right),$$

where $g=\bar{1,G^{**}}$.

The data rate $V_{g}^{**}\left(R^{s}\right)$ calculating method, depending on the reception algorithm $R^{S}$ is discussed in [8].

Values $V_{g}^{opt**}$ and $R_{g}^{S\_opt**}$ obtained based on expressions (7) and (8) form data rate vectors ${\vec{V}}^{DU}$, ${\vec{V}}^{UU}$, and ${\vec{V}}^{DS}$, ${\vec{V}}^{US}$, as well as reception algorithms vectors ${\vec{R}}^{SDU}$,${\vec{R}}^{SUU}$, and ${\vec{R}}^{SDS}$, ${\vec{R}}^{SUS}$, respectively:

(9) $${\vec{V}}^{**}=\left(V_{1}^{opt**},\ldots ,V_{G^{**}}^{opt**}\right),$$

(10) $${\vec{R}}^{S**}=\left(R_{1}^{S\_opt**},\ldots ,R_{G^{**}}^{S\_opt**}\right).$$

This operation is performed at the signal reception procedure analysis stage using the signals reception procedure analysis function $F^{ra}$:

(11) $$\left(\;{\vec{V}}^{**},{\vec{R}}^{S**}\right)=F^{ra}\left(\;\left\{w^{**}\right\},{\vec{V}}^{pd**},\left\|{\tilde{H}}^{**}\right\|,\left\|{\tilde{\tau }}^{**}\right\|\right).$$

The work [8] provides an effectiveness analysis of the developed reception algorithm based on the optimal measurement method [22] in comparison with the currently widely used reception algorithm based on the Fast Fourier Transform (FFT). It is shown that the optimal measurement algorithm of the company ZAO Torus-Volga Wi-Fi network segment allows for an increase in data rate of up to two times compared to the standard FFT-based reception algorithm by reducing the intra-system interference influence at the adjacent channel by approximately 12% of seats at the stadium in Kaliningrad (Russian Federation). At the same time, at other the considered section points of the stadium’s stands, characterized by an insignificant intra-system interference level at the adjacent channel, the channel data rates of both algorithms are comparable. However, due to the complexity of implementing the developed algorithm on the users and sensor equipment, this algorithm will only be applied to APs.

Next, the real volumes of data delivered $\overset{\vec{⌢}}{I}\overset{**}{⠀}$ and network load $A^{net**}$ upon delivery of the required volumes of data being delivered ${\vec{I}}^{\max**}$ are calculated. These values are determined at the routing procedure analysis stage:

(12) $$\left(\overset{\vec{⌢}}{I}\overset{**}{⠀},A^{net**}\right)=F^{wi}\left(\;{\vec{I}}^{\max**},{\vec{V}}^{**}\right),$$

where $F^{wi}$ is the routing procedure function for calculating the real volumes of data delivered and the network load.

The value $A^{net**}$ is defined as the ratio of the time required to deliver the required volumes of data being delivered ${\vec{I}}^{\max**}$ to the information accumulation interval $T^{I}$ ($T^{S}$). When calculating the real volumes of data delivered $\overset{\vec{⌢}}{I}\overset{**}{⠀}$, it is assumed that $\overset{\vec{⌢}}{I}\overset{**}{⠀}=\gamma \cdot {\vec{I}}^{\max**}$, where γ is determined from the condition $A^{net}=A^{\max}$.

The received information is processed at the FTP procedure dynamic planning stage in order to minimize $A^{net}$, and therefore an increase of $\vec{\overset{⌢}{I}}=\left(\overset{\vec{⌢}}{I}\overset{DU}{⠀},\overset{\vec{⌢}}{I}\overset{UU}{⠀},\overset{\vec{⌢}}{I}\overset{DS}{⠀},\overset{\vec{⌢}}{I}\overset{US}{⠀}\right)$.

Next, the obtained minimum values $A^{net**}$ are used at the FTP procedure static planning stage, where the current network parameter's compliance with the requirements for it is assessed. If the requirements are not met, the static planning parameters vector ${\vec{V}}^{ps}$ is corrected, according to expression (1), and the planning and optimizing network parameters process, according to expressions (1), (4)–(12), is repeated until the requirements are not met.

When the requirements for the network are met, the network is ready to transmit information. Data vectors with current volumes of data being delivered ${\vec{I}}^{DU}=\left(I_{1}^{DU},\ldots ,I_{N^{U}}^{DU}\right)$, ${\vec{I}}^{UU}=\left(I_{1}^{UU},\ldots ,I_{N^{U}}^{UU}\right)$, ${\vec{I}}^{DS}=\left(I_{1}^{DS},\ldots ,I_{N^{S}}^{DS}\right)$, ${\vec{I}}^{US}=\left(I_{1}^{US},\ldots ,I_{N^{S}}^{US}\right)$ are received as input to the routing procedure. To transmit incoming data in the routing procedure, at the routing stage, multidimensional routes are recurrently formed. In [6], for a seamless Wi-Fi network with sensors, a modified data transmission structure in the CDR method was developed. It allows for reducing the sensors' energy consumption while maintaining high data rates and short data delivery times for mobile users of the seamless IEEE 802.11ax network.

However, work [6] does not take into account the battery charge $C_{n^{S}}^{S}$ for sensors, which is important for increasing the network lifetime [23]. If $C_{n^{S}}^{S}$ is equal to 0, then no data is transmitted (received) from such a device. If $C_{n^{S}}^{S}$ is equal to 1, then such a sensor does not have priority when transmitting (receiving) data. Reducing battery power $C_{n^{S}}^{S}$ leads to the fact that the data from such a sensor must be transmitted first. Since the multidimensional route formation is carried out according to minimizing the delivery time criterion, to take into account such a sensor priority instead of its frame duration $T^{FS}$, according to [6], the ratio $T^{FS}/C_{n^{S}}^{S}$ is used. Reducing the battery charge $C_{n^{S}}^{S}$ leads to an increase in the ratio $T^{FS}/C_{n^{S}}^{S}$, to increase sensor priority.

When using a robust method for estimating the channel data rate, the construction of multidimensional routes is carried out sequentially, starting with the user (sensor), to which the maximum data volume must be transmitted (received) [6]. In this case, such a user (sensor) uses one-dimensional routes that provide maximum data rates. Subsequent users (sensors) use the remaining one-dimensional routes with lower data rates to transmit data. Therefore, to take into account the sensor battery charge influence by using a robust method, it is proposed to increase its priority by initially searching in the vector ${\vec{I}}^{*S}$ of sensor $n_{*}^{S}$ with the maximum value of the quantity $I_{n^{S}}^{*S}/C_{n^{S}}^{S}$.

Thus, to transmit data with volumes $\vec{I}$ at the routing stage, multidimensional routes are recurrently formed based on the minimizing data delivery time criterion:

(13) $$w_{i}^{M}=F^{w}\left(\vec{I},\vec{V},{\vec{C}}^{S},{\vec{N}}^{RU},i\right),$$

where $F^{w}$ is the routing procedure function for multidimensional routes recurrent formation, $\vec{V}=\left({\vec{V}}^{DU},{\vec{V}}^{UU},{\vec{V}}^{DS},{\vec{V}}^{US}\right)$ is the data rates vector, and i is the frame number.

According to the work [6], for a seamless Wi-Fi network with sensors, multidimensional routes are formed separately to transmit data between BR and users and BR and sensors.

Based on each generated multidimensional route, data streams are generated that arrive at the transmitting nodes, taking into account the information contained in the vectors ${\vec{V}}^{pd**}$.

These transmitted signals are received in the signal reception procedure, and the transfer $\left\|\tilde{H}\right\|$ and delay $\left\|\tilde{\tau }\right\|$ matrices estimates are corrected:

(14) $$\left(\;\left\|{\tilde{H}}^{**}\right\|,\left\|{\tilde{\tau }}^{**}\right\|\right)=F^{r}\left(\;\left\{w^{**}\right\},{\vec{V}}^{pd**},\left\|{\tilde{H}}^{**}\right\|,\left\|{\tilde{\tau }}^{**}\right\|\right),$$

where $F^{r}$ is the data reception function of the signal reception procedure.

The complex transmission coefficient and the data transmission delay over the CC for each of the receiving nodes are estimated using standard tools implemented on all Wi-Fi adapters. When the received estimates change, the node sends these estimates to the BR, where $\left\|\tilde{H}\right\|$ and $\left\|\tilde{\tau }\right\|$ matrixes are formed for the entire network. Values $\left\|\tilde{H}\right\|$ and $\left\|\tilde{\tau }\right\|$ are further used in the signals reception procedure and at the FTP procedure in dynamic planning stage to correct vector ${\vec{V}}^{pd**}$.

From the above expressions it follows that in order to ensure a comprehensive reduction in the intra-system interference influence, the considered procedures must interact with each other. To ensure their interaction, a unified parameters set $V^{u}$ is used. It includes the parameters of all procedures.

At the same time, it should be taken into account that the parameter change rates included in the unified parameters set vary significantly. The analysis of these differences allowed to separate the parameters included in the unified set $V^{u}$, according to their change rate, into three groups:

1. Fixed parameters are constant during the network operation and change only when its configuration changes. These include the required volumes of data delivered ${\vec{I}}^{\max}$ and the static network planning parameters vector ${\vec{V}}^{ps}$.
2. Variable parameters, which change rate, are due to significant changes in device parameters (sensor battery charge ${\vec{C}}^{S}$), as well as CC parameters in the form of transfer $\left\|\tilde{H}\right\|$ and delay $\left\|\tilde{\tau }\right\|$ matrices estimates. Their formation, due to the randomness of traffic transmitted over the network, is carried out over a time interval that includes a sufficient number of data accumulation intervals $T^{I}$. These include one-dimensional routes subsets $\left\{w^{**}\right\}$, dynamic planning parameter vectors ${\vec{V}}^{pd**}$, and used reception algorithms vectors ${\vec{R}}^{S}=\left({\vec{R}}^{SDU},{\vec{R}}^{SUU},{\vec{R}}^{SDS},{\vec{R}}^{SUS}\right)$ determined on their basis, data rates vector $\vec{V}$, as well as the real volumes of data delivered $\vec{\overset{⌢}{I}}$ and network load $A^{net}$.
3. Traffic parameters, the values of which are determined by a random data flow at each data accumulation interval $T^{I}$. These include the current volumes of data being delivered $\vec{I}$ and multidimensional routes $w_{i}^{M}$ determined on their basis.

In connection with the above separation of parameters according to their change rate, optimization is carried out for each of the groups separately, and the groups’ parameters with a slower change parameters rate are considered known during optimization, and the group's parameters with a high change rate are chosen as the worst. In this case, the unified parameters set will take the following form:

(15) $$V^{u}=\left({\vec{I}}^{\max},{\vec{V}}^{ps};\left\|\tilde{H}\right\|,\left\|\tilde{\tau }\right\|,\left\{w^{DU}\right\},\left\{w^{UU}\right\},\left\{w^{DS}\right\},\left\{w^{US}\right\},{\vec{V}}^{pd},{\vec{R}}^{S},\vec{V},\vec{\overset{⌢}{I}},A^{net},{\vec{C}}^{S};\vec{I},w_{i}^{M}\right).$$

Since the parameters included in the unified set ${\vec{V}}^{u}$ are calculated in some procedures and, at the same time, are input parameters for other procedures, then their calculation must be performed in a certain order. Thus, IOM is a sequence of interrelated actions aimed at improving network performance by comprehensively reducing the impact of intra-system interference.

## 5. Algorithm for a Novel Modification of the Integrated Optimization Method for Wi-Fi Networks with Sensors

**Figure 3:**
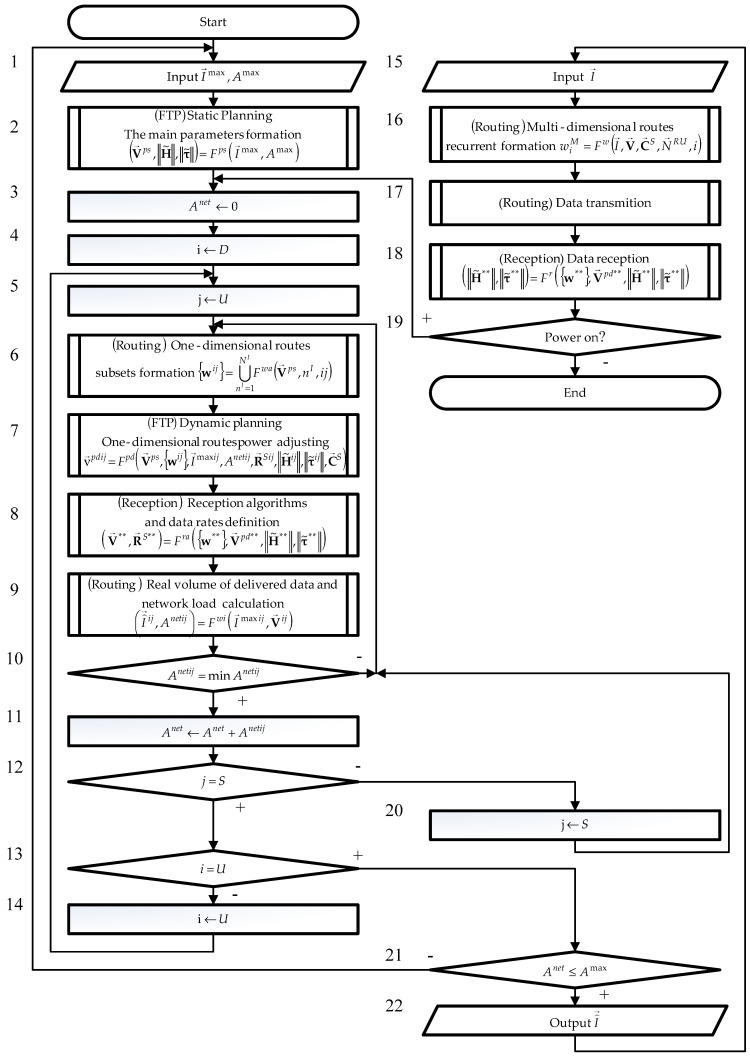
The operating algorithm block diagram of a novel modification of IOM for seamless Wi-Fi networks with sensors.

To describe in detail the sequence of these actions, an algorithm for the operation of a novel modification of IOM for Wi-Fi networks with sensors was developed. Its block diagram is shown in Figure 3.

In the algorithm, the belonging of blocks to the procedures for signal reception, FTP, and routing is indicated by the expressions Reception, FTP, and Routing, respectively.

Initially, in block 2, static planning is carried out. Its task is to form the basic SIE parameters that ensure the required volumes of data being delivered ${\vec{I}}^{\max}$ delivery, entered in block 1, by specifying a static planning parameters vector ${\vec{V}}^{ps}$ and generating transfer $\left\|\tilde{H}\right\|$ and delay $\left\|\tilde{\tau }\right\|$ matrices estimates.

In block 3, the network load value $A^{net}$ is set to 0. In block 4, variables i are assigned as D (downlink), and in block 5, the variable j is assigned as U, which corresponds to the data transmission mode to users.

Vector-based ${\vec{V}}^{ps}$ in the routing procedure (block 6) subsets of one-dimensional routes are formed for all APs $\left\{w^{ij}\right\}=\overset{N^{I}}{\underset{n^{I}=1}{\cup }}\left\{w_{n^{I}}^{ij}\right\}$.

Next, in block 7, dynamic planning is carried out. Its purpose is to optimize SIE parameters for each one-dimensional route, allowing for minimization of the load on the network by reducing the intra-system interference influence.

Based on information about SIE for each one-dimensional route in the reception procedure (block 8), reception algorithms ${\vec{R}}^{Sij}$ are selected, ensuring maximum data rate by effectively combating intra-system interference.

Further, in block 9, in the routing procedure based on the data rate vector ${\vec{V}}^{ij}$ , the real volumes of data delivered ${\vec{\overset{⌢}{I}}}^{ij}$ and network load $A^{netij}$ are determined.

Minimizing value $A^{netij}$ is carried out through the joint use of routing, dynamic planning, and signal reception procedures in blocks 6–9. Finding the minimum $A^{netij}$ is controlled in block 10.

A minimum value $A^{netij}$ is added in block 11 to the load $A^{net}$.

Next, in block 12, the variable j is checked. If it does not match the value S (data transmission to sensor), then in block 20, the variable j is assigned as S , and the transition to block 6 is carried out, i.e., the value $A^{netij}$ is minimized for the data transmission to sensors. Otherwise, the variable i in block 13 is checked.

If it does not match the value U (uplink), then in block 14, the variable i is assigned U , and the transition to block 5 is carried out, i.e., the value $A^{netij}$ is minimized in the uplink. Otherwise, the resulting value $A^{net}$ is compared with the maximum permissible network load $A^{\max}$ in block 21. If the network load $A^{net}$ is less than or equal to the acceptable value $A^{\max}$, then a frequency–territorial plan for a seamless Wi-Fi network with sensors and a unified parameters set $V^{u}$ is formed. Thus, the network is ready to transmit data. Otherwise, you need to go back to the static planning stage (block 1).

Based on the parameters included in the unified set $V^{u}$, in the routing procedure (block 16), according to the minimizing delivery time criterion for the current volume of data being delivered $\vec{I}$, entered in block 15, multidimensional routes are formed.

After the multidimensional routes formation, in block 17, the procedure for transmitting data along the generated multidimensional routes is started, and in block 18, the procedure for receiving data is started.

At the same time, based on the data delivery results carried out in blocks 17 and 18, the CC parameters are corrected.

When power is available to maintain optimal SIE settings and minimize network load $A^{net}$ in block 7, the network dynamic planning is carried out again, affecting data rates. The data rates updated in block 8 will be taken into account when transmitting the next data in the routing procedure.

Thus, a comprehensive reduction in the intra-system interference influence is carried out, including reducing the interference flow in the routing procedure, effectively combating intra-system interference in the signals reception procedure by choosing a reception algorithm, and reducing the intra-system interference influence on the selected reception algorithm in FTP procedure by controlling SIE.

Since the vector ${\vec{V}}^{ps}$ defined in block 2 is fixed in the frequency–territorial plan, its change without special approval is not allowed. Therefore, automatic function $F^{ps}$ start is not possible.

Other parameters included in the set ${\vec{V}}^{u}$ may change during the network operation.

A variable parameters group is associated with functions of the dynamic planning stage $F^{pd}$, the signal reception analysis stage $F^{ra}$, and routing procedures $F^{wa}$ and $F^{wi}$. Their task is to optimize variable network parameters in order to minimize the network load $A^{net}$ when the CCs or device parameters are changed. Since the listed function parameters have a unique dependence on each other, they must be executed sequentially: $F^{wa}$, $F^{pd}$, $F^{ra}$, and $F^{wi}$(blocks 6–9). The listed functions set forms the IOM optimization process, which should be separated into a separate algorithm branch.

The current volumes of data being delivered $\vec{I}$ and the multidimensional routes $w_{i}^{M}$ determined on their basis are included in the traffic parameters group and are used by the routing procedure's main function $F^{w}$ (block 16).

CC’s parameters $\left\|\tilde{H}\right\|$ and $\left\|\tilde{\tau }\right\|$ are evaluated in the signals reception procedure (block 18) during data transmission.

Since the transmitting and receiving nodes are physically separated, and the change rates in groups of variable parameters and traffic parameters are significantly different, the processes of data transmission (blocks 16–17), data reception (block 18), and network parameters optimization (blocks 6–9) must be performed parallel. The algorithm for transmitting data in IOM IP networks is given in [21]. In this case, blocks 3–16 and 20–22 are executed on the BR, and blocks 17 and 18 are executed on the receiving and transmitting nodes, respectively.

In real-time, only forming multidimensional routes, transmitting and receiving data are performed (blocks 16–18). The transmitting and receiving data using an FFT-based algorithm are implemented in existing equipment. The resources required to implement the optimal measurement algorithm are given in [22].

## 6. Evaluation of Novel Modification of Integrated Optimization Method Effectiveness

**Figure 4:**
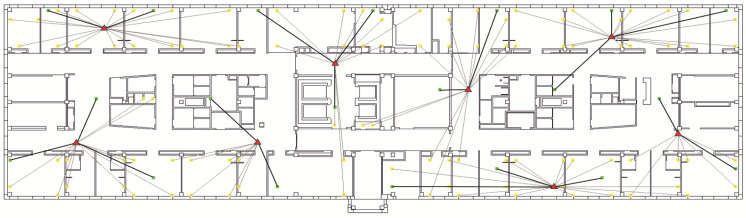
Location of APs, users, and sensors of the seamless Wi-Fi network segment [6].

The effectiveness of using the novel modification of IOM in comparison with the novel modification of the CDR method was evaluated on the seamless IEEE 802.11ax network segment, considered in [6]. The Wi-Fi network segment consists of eight Eltex WEP-3ax APs operating on 1, 6, and 11 frequency channels in a 2.4 GHz frequency range with a 20 MHz band. The network is designed to serve 20 users and 100 fixed sensors based on the ESP32-C6 processor [24]. Wi-Fi network segment simulation was carried out at a fixed point in time. The location of APs, users, and sensors at this moment is shown in Figure 4.

In Figure 4, the AP locations are indicated by red triangles, and users and sensors are indicated by green and yellow squares, respectively. The users' and sensors’ connection to APs is indicated in Figure 4 by thick and thin black lines, respectively.

When modeling a Wi-Fi network segment using the OFDM Planning software 2023 package, it was assumed that HTTP/TCP and FTP protocol packets generated using the 4IPP model [25] were transmitted to (from) all users. Traffic rates for all users were considered the same and ranged from 1 to 10 Mbit/s. The information vector accumulation interval duration for users $T^{I}$ , according to [6], was chosen to be equal to 20 ms. Packets transmitted by sensors were generated according to the Pareto model [26], provided that the average data delivered volume to the sensors in 1 s was 100 bytes, and from the sensors 1 kB. The total analysis time was 100 s.

The sensors were divided into 50 groups, and at each interval $T^{I}$, two sensors were serviced. Since the analysis time was only 100 s, to obtain a clear change in the battery charge, its capacity was chosen to be 50 μA/h. The data frame duration to (from) users was $T^{FA}$ = 720.8 μs, and the data frame duration to (from) the sensors was $T^{FS}$ = 190.4 μs. Initially, the APs and users' radiated powers were considered equal to 19 dBm, and the sensors radiated power was considered to be 0 dBm.

Based on the simulation results, the network load $A^{net}$, data rate over the network as a whole for users $V^{\Sigma U}$ and sensors $V^{\Sigma S}$, and dependence of sensor battery charge on network operating time were determined.

In this work, the network load $A^{net}$ was determined as in [6]:

(16) $$A^{net}=\frac{1}{T^{S}}\cdot \left(T^{FU}\cdot \left(\sum_{n^{SP}=1}^{N^{SP}}N_{n^{SP}}^{FU}+1\right)+T^{FS}\cdot \sum_{n^{SP}=1}^{N^{SP}}N_{n^{SP}}^{FS}\right),$$

where $N_{n^{SP}}^{FU}$ is the number of frames during which data was transmitted to (from) users, $N_{n^{SP}}^{FS}$ is the number of frames during which data was transmitted to (from) sensors to $n^{SP}$-th information accumulation interval $T^{I}$.

**Figure 5:**
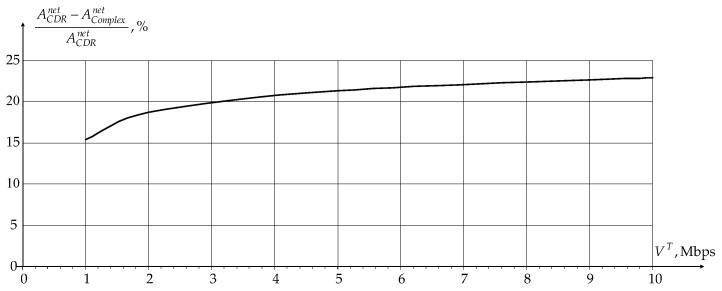
Dependence of gain in network load on user’s traffic rate as a percentage.

**Figure 6:**
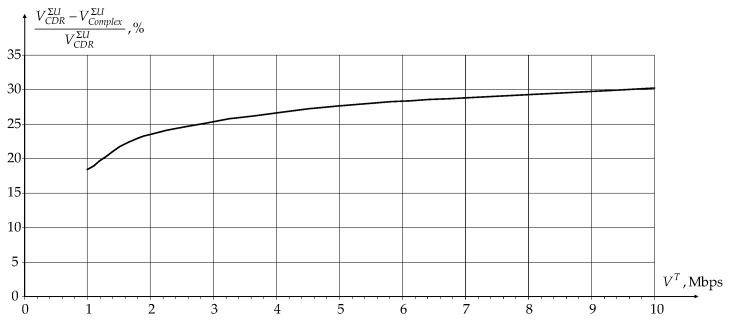
Dependence of the gain in data rate over the network as a whole for users on the traffic rate as a percentage.

Since multidimensional routes are formed separately for data transmission between BR and users and BR and sensors, the application of a novel modification of IOM for users was first analyzed at first. Figure 5 and Figure 6 show the dependence gain in network load and data rate over the network as a whole for users compared to the novel modification of the CDR method as a percentage of the user traffic rate $T^{S}$ equal to 1.0 s, respectively.

From the graphs shown in Figure 5 and Figure 6, it can be seen that the use of a novel modification of IOM allows for reducing the network load $A^{net}$ and increases the data rate over the network as a whole for users $V^{\Sigma U}$ due to changing the radiated power of APs and users in the dynamic planning procedure and the use of an optimal measurement algorithm in the signals reception procedure on APs. Thus, the joint use of routing, dynamic planning, and signal reception procedures makes it possible to comprehensively reduce the intra-system interference influence and, thereby, improve the network characteristics.

The main effectiveness indicators of using a novel modification of IOM for accessing wireless sensors are the data rate over the network as a whole for sensors $V^{\Sigma S}$ and sensor lifetime. As a simulation result, the gain in data rate over the network as a whole for sensors $V^{\Sigma S}$ compared to the CDR method was 40%. This is due to changes in the radiated power of APs and sensors in the dynamic planning procedure, as well as the use of an optimal measurement algorithm in the signals reception procedure on APs.

**Figure 7:**
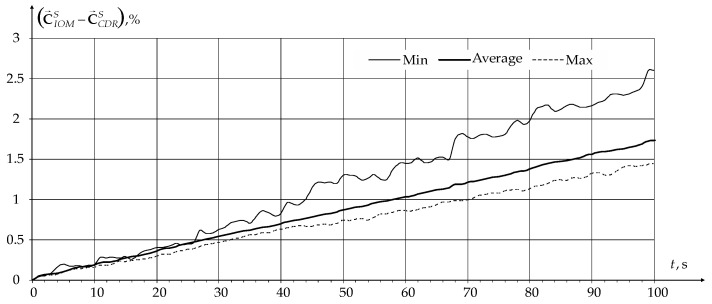
Dependencies of the gain in sensor battery charge for the novel modification of IOM compared to the novel modification of the CDR method as a percentage.

To estimate the sensor's lifetime, battery charge values were calculated during the simulation process ${\vec{C}}^{S}$ for all network sensors at each interval $T^{S}$. Based on the data obtained at each $T^{S}$ minimum $\min\left\{{\vec{C}}^{S}\right\}$, average $\bar{{\vec{C}}^{S}}$ , and maximum $\max\left\{{\vec{C}}^{S}\right\}$ battery charge values among all network sensors were determined. The dependences of the gain in the obtained values for the novel modification of IOM compared to the novel modification of the CDR method on the network operating time as a percentage are shown in Figure 7.

From the graphs shown in Figure 7, it can be seen that in the data transmission process, the maximum gain in sensor battery charge is achieved for sensors with a minimum battery charge level due to comprehensive network parameters optimization. This leads to an increase in network operation time with all sensors.

**Figure 8:**
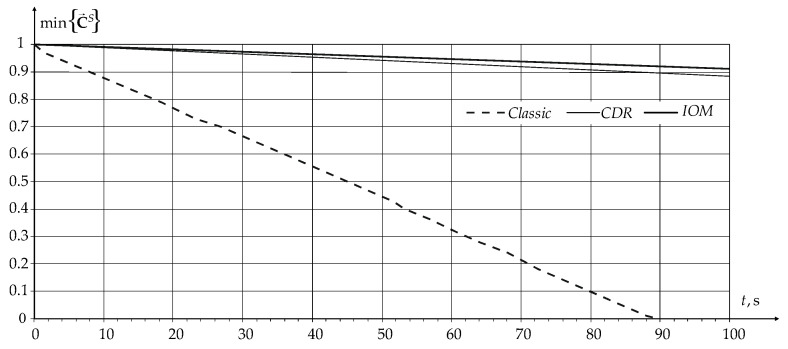
Dependence of the minimum value of the sensor battery charge on the network operating time.

Therefore, to estimate the sensors’ lifetime, the dependences of the minimum sensors’ battery charge on the network operating time were plotted for the cases of connecting the sensors to a standard Wi-Fi network, as well as when using a novel modification of the CDR method and a novel modification of IOM, which are shown in Figure 8.

From the dependencies shown in Figure 8, it follows that the use of a novel modification of the CDR method and a novel modification of IOM makes it possible to reduce the sensor battery's discharge rate. When installing 1000 mAh batteries on the sensors, the average sensor lifetime will be only 19 days in a standard Wi-Fi network, 160 days when using the novel modification of the CDR method, and 193 days when using the novel IOM modification.

## 7. Conclusions

A novel modification of IOM for heterogeneous networks using the seamless Wi-Fi networks example simultaneously serving mobile users and wireless sensors has been developed in this work. Based on the mutual influence analysis of procedures for signal reception, FTP, and routing in heterogeneous networks that provide simultaneous data transmission by mobile users and sensors, new principles for the interaction of these procedures are formulated, basic functions are obtained to describe the interaction of procedures, a novel modification of IOM is developed, and the algorithm for its operation.

For the IEEE 802.11ax network segment considered in the work, the use of a novel modification of IOM made it possible to reduce the network load by an average of 20%, increase the data rate over the network as a whole for users and sensors by an average of 25% and 40%, respectively, and also increase the lifetime of sensors is on average 20% compared to the novel modification of CDR method.

## Data Availability

No new data were created or analyzed in this study. Data sharing is not applicable to this article.
